# Whole genome sequence of the syntrophic benzoate-degrading bacterium, *Syntrophus buswellii* DSM 102354^**T**^

**DOI:** 10.1128/mra.00266-25

**Published:** 2025-07-31

**Authors:** Robert P. Gunsalus, Ethan Humm, Lauren Cook, Neil Q. Wofford, Kimberly L. James, Matteo Pellegrini, Marco Morselli, Sung Min Ha, Michael J. McInerney

**Affiliations:** 1 Microbiology, Immunology and Molecular Genetics, University of California 8783, Los Angeles, California, USA; 2 DOE Institute, University of California 8783, Los Angeles, California, USA; 3 Microbiology and Plant Biology, University of Oklahoma 6187, Norman, Oklahoma, USA; 4 Molecular, Cell and Developmental Biology, University of California 8783, Los Angeles, California, USA; 5 Integrative Biology and Physiology, University of California 8783, Los Angeles, California, USA; California State University San Marcos, San Marcos, California, USA

**Keywords:** *Syntrophus*, genome analysis, syntrophs, aromatic compounds

## Abstract

The syntrophic obligate proton-reducing bacterium *Syntrophus buswellii* DSM 102354^T^, isolated from anaerobic digester sludge, degrades benzoate and possibly hydrocinnamate (phenyl-3-propionate) when grown with a suitable hydrogenotrophic partner. The complete genome sequence data provide insight regarding aromatic compound degradation under thermodynamically limiting conditions when exogenous electron acceptors are absent.

## ANNOUNCEMENT

The genus *Syntrophus* belongs to the family Syntrophaceae and members live in many natural and man-made anaerobic habitats where they participate in anaerobic biomass recycling (1–3). *Syntrophus buswellii* DSM 102354^T^ (=DSM 2612M^T^) was isolated from a tri-culture enrichment obtained from the DSMZ German Collection (DSM 2612B^T^) by serial dilution using the Hungate technique and a bicarbonate-buffered mineral-salts medium containing crotonate as the sole carbon supply (4). Taxonomic identification was carried out by PCR-based 16S rRNA gene sequencing (EMBL data library accession number X85131) and subsequent comparative analysis with 1,600 bacterial 16S rRNA reference sequences (4). The above-described *S. buswellii* DSM 2612B^T^ tri-culture enrichment was previously derived from an anaerobically collected sewage sample from the primary anaerobic digester at the Urbana, Illinois, waste digestion facility (40.109 N, 88.204 W) prior to 1982 (5, 6). When *S. buswellii* DSM 102354^T^ is cultured syntrophically with a suitable hydrogenotrophic partner such as *Methanospirillum hungatei* JF-1^T^, it metabolizes benzoate and presumably other aromatic substrates (2, 7).

*S. buswellii* DSM 102354^T^ was obtained from the McInerney laboratory culture collection and cultivated anaerobically in a mineral-salts medium using the Hungate method with 20 mM crotonate as the sole carbon supply (3, 7). Cells were grown statically at 37°C to late log phase, harvested anaerobically, and stored at −70°C until processing. DNA (15.3 ng/µl) was extracted from cell pellets using the Zymo D4081 Quick-DNA Magbead Plus Kit as per the manufacturer’s instructions. DNA was then fragmented using Covaris g-Tubes in an Eppendorf 5424 centrifuge for 60 s at 3,000 g for a total of 3 spins according to the manufacturer’s protocol. The average size of the sheared DNA was then calculated using the genomic DNA Assay (TapeStation, Agilent Technologies) with an average of 12.9 Kb (median = 12.4 Kb, min = 10.4 Kb, and max = 19.8 Kb) for the construction of genomic libraries. Sheared DNA was then concentrated using AMPure PB beads according to the manufacturer’s recommendations (PacBio—multiplexed microbial libraries using SMRTbellExpress Template Prep Kit 2.0, #101-696-100 version 07, July 2020). The purified sheared DNA had an average size of 11.8 Kb (median = 11.7 Kb, min = 9.5 Kb, and max = 14.7 Kb) and a concentration of 74.4 ng/µl. Library preparation was performed according to the PacBio protocol. DNA sequencing was performed using the PacBio Sequel IIe platform, which generated 62,884 raw reads. Demultiplexing and adapter trimming were done using Lima v2.9.0 (https://github.com/pacificbiosciences/barcoding). All reads were then targeted for assembly by Canu v2.2 (8). Assembled genomes were further refined by Circlator v1.5.5 (9) to identify circular contigs, remove redundant non-circular contigs, and rotate circular contigs to start with *dnaA*. A completeness check was performed by CheckM v1.0.18 (10), and the N50 quality was determined by Assembly stats v1.01 (https://github.com/sanger-pathogens/assembly-stats). Genome ORF calling and annotation were performed by NCBI PGAP v6.9 (11).

The properties of the circular *S. buswellii* DSM 102354^T^ genome are shown in Table 1.

**TABLE 1:** *S. buswellii* DSM 102354T genome information

Feature	Genome value
Topology	Circular
Size (bp)	3,252,431
GC%	55.23%
Protein coding genes	2,834
16S number	1
tRNA number	48
Coverage	35.58×
Raw reads	62,884
Average read length	8,186 bp
High-quality reads	9,290
N50 quality value (bp)	3,252,431
Completeness value	97.1%
16S similarity^*a*^	99.62%
Sample collection site	40.109 N, 88.204 W
NCBI taxonomy ID	43774

^*a*^ 16S similarity to the published 16S accession no. X85131 determined by NCBI blastn (12).

## GENOME HIGHLIGHTS

The genome contains two paralogs each of benzoyl-CoA ligase and benzoyl-CoA reductase for aromatic ring activation and reduction, suggesting the ability to degrade additional aromatic compounds besides benzoate (3, 13). It lacks pta and ack genes for substrate-level ATP formation from acetyl-CoA and instead contains AMP-forming acetyl-CoA synthetase genes for ATP synthesis as demonstrated in *Syntrophus aciditrophicus* (14).

## Data Availability

The complete genome sequence of *Syntrophus buswellii* DSM 102354T has been deposited in the GenBank assembly accession GCA_046097065.1, and the raw sequencing reads have been deposited under the accession no. SRR31591383. The IMG genome OID number is 8025829216 and the IMG submission ID is 303293.
